# Nuclear Insulin-Like Growth Factor Binding Protein-3 As a Biomarker in Triple-Negative Breast Cancer Xenograft Tumors: Effect of Targeted Therapy and Comparison With Chemotherapy

**DOI:** 10.3389/fendo.2018.00120

**Published:** 2018-03-22

**Authors:** Sohel M. Julovi, Janet L. Martin, Robert C. Baxter

**Affiliations:** Kolling Institute, University of Sydney, Royal North Shore Hospital, St. Leonards, Sydney, NSW, Australia

**Keywords:** insulin-like growth factor binding protein-3, basal-like, triple-negative, breast cancer, targeted therapy, chemotherapy

## Abstract

Triple-negative breast cancer (TNBC) typically has a worse outcome than other breast cancer subtypes, in part owing to a lack of approved therapeutic targets or prognostic markers. We have previously described an oncogenic pathway in basal-like TNBC cells, initiated by insulin-like growth factor binding protein-3 (IGFBP-3), in which the epidermal growth factor receptor (EGFR) is transactivated by sphingosine-1-phosphate (S1P) resulting from sphingosine kinase (SphK)-1 activation. Oncogenic IGFBP-3 signaling can be targeted by combination treatment with the S1P receptor modulator and SphK inhibitor, fingolimod, and the EGFR kinase inhibitor, gefitinib (F + G). However, the interaction of this treatment with chemotherapy has not been documented. Since we observed nuclear localization of IGFBP-3 in some TNBC tumors, this study aimed to evaluate the prognostic significance of nuclear IGFBP-3 in pre-clinical models of basal-like TNBC treated with F + G and doxorubicin. Orthotopic xenograft tumors were grown in nude mice from the human basal-like TNBC cell lines MDA-MB-468 and HCC1806, and were treated with gefitinib, 25 mg/Kg, plus fingolimod, 5 mg/Kg, 3-times weekly. In some studies, doxorubicin was also administered once weekly for 6 weeks. Tumor tissue proteins were quantitated by immunohistochemistry (IHC). Interaction between doxorubicin and F + G was also studied in proliferation assays *in vitro*. In both tumor models, tissue staining for IGFBP-3 was predominantly nuclear. Combination of F + G significantly enhanced mouse survival, decreased nuclear IGFBP-3 and Ki67 staining, and increased apoptosis (cleaved caspase-3) staining. Kaplan–Meier survival analysis showed that a high tumor IGFBP-3 IHC score (>median), like a high Ki67 score, was significantly associated with shorter survival time, whereas a high apoptosis score was associated with prolonged survival. Studied *in vitro* in both cell lines, low-dose doxorubicin that had little effect alone, strongly enhanced the cytostatic effect of low-dose F + G combination. However, in both *in vivo* models, doxorubicin at maximum-tolerated dose neither inhibited tumor growth when administered alone, nor enhanced the significant inhibitory effect of F + G. We conclude that doxorubicin may not add benefit to the inhibitory effect of F + G unless its dose-limiting toxicity can be overcome. Nuclear IGFBP-3 appears to have potential as a prognostic marker in TNBC and could be evaluated for clinical utility.

## Introduction

Breast cancer is recognized as a heterogeneous disease, commonly classified into subtypes on the basis of phenotypic and/or molecular characteristics (1, 2). Of the breast cancers with absent or very low estrogen receptor (ER) and progesterone receptor (PR), and without overexpression of the human epidermal growth factor receptor-2, known as triple-negative breast cancers (TNBC), about 80% have a basal-like profile, in terms of both gene expression profile (3) and the display of established basal histology markers, such as cytokeratin 5/6 and the epidermal growth factor receptor (EGFR) (4). Triple-negative breast cell lines that display a basal-like phenotype and have high EGFR expression (5) include MDA-MB-468 (classified as basal-like 1) and HCC1806 (classified as basal-like 2) (3).

There are no established molecular targets or prognostic markers for TNBC, which typically has a worse outcome than other breast cancer subtypes (6). Immunotherapeutic approaches have recently shown some durable responses, and the development of tumor-specific neoantigens and other new therapeutic targets offers hope for the future (7). Our recent pre-clinical studies have revealed an unexpected immune modulator in a murine TNBC-like model, with the demonstration that mammary tumors in mice null for insulin-like growth factor binding protein-3 (IGFBP-3) grow 50% smaller than those in wild-type mice and show increased accumulation of CD8^+^ lymphocytes (8). In wild-type mice, tumor IGFBP-3 gene expression was positively associated with tumor weight (8), consistent with some clinical studies showing that high IGFBP-3 abundance in breast tumor tissue is associated with high tumor grade (9) and poor prognosis (10, 11).

Insulin-like growth factor binding protein-3 is also known to drive an oncogenic pathway in human TNBC cell lines involving activation of the receptor tyrosine kinase, EGFR, and the lipid kinase, sphingosine kinase (SphK) (12, 13). We have shown that inhibitors of these two IGFBP-3-activated kinases, administered in combination, act synergistically to significantly inhibit TNBC cell growth *in vitro* and in xenograft tumors (5, 13). However, the importance of high tumor IGFBP-3 levels in the progression of many cancer types remains unclear because there are some cancers in which *IGFBP3* appears to act as a tumor suppressor gene, with *low* IGFBP-3 levels associated with poor patient outcome (14).

Insulin-like growth factor binding protein-3, a secreted glycoprotein found in both the circulation and the pericellular/intracellular environment, is known to translocate to the cell nucleus in some conditions, and its interaction with nuclear ligands, influencing both gene transcription and DNA damage repair, has been documented (15). Intriguingly, while nuclear interactions of IGFBP-3 have been associated with its induction of apoptotic death in prostate cancer cell lines (16, 17), a clinical study showed that high nuclear staining of IGFBP-3 in prostate cancer tissue was prognostic for earlier disease recurrence (18).

In breast cancer, the significance of nuclear IGFBP-3, both functionally and as a biomarker, is not fully understood. The primary goal of this study was to evaluate the prognostic significance of nuclear IGFBP-3 using pre-clinical models of basal-like TNBC treated with EGFR and SphK inhibitors. We also examined the relationship between nuclear IGFBP-3 and indicators of tumor proliferation and apoptosis. Our secondary goal was to compare combination kinase inhibition with the chemotherapeutic agent doxorubicin *in vitro* and *in vivo*, and to evaluate the effect of co-administering the targeted drugs with chemotherapy.

## Materials and Methods

### Reagents

The EGFR kinase inhibitor gefitinib (ZD1838, Iressa), the SphK inhibitor and sphingosine-1-phosphate receptor modulator, fingolimod (FTY720, Gilenya), and the chemotherapy drug doxorubicin (adriamycin), were purchased from MedChem Express, Princeton, NJ USA.

### Human TNBC Cell Lines

The human basal-like TNBC cell lines, MDA-MB-468 and HCC1806, were obtained from ATCC, Manassas, VA, USA and maintained under standard conditions in RPMI 1640 medium containing 5% FBS and 10 µg/mL bovine insulin. Stocks of these cells were frozen within 1 month of purchase, and fresh cultures for experimental use were established every 2–3 months. All cell lines were negative on mycoplasma testing.

### Measurement of Cell Proliferation

Cells (4 × 10^3^/well for HCC1806; 8 × 10^3^/well for MDA-MB-468) were dispensed into 96-well plates in complete medium and incubated overnight before changing to fresh medium containing 5% FBS and inhibitors. The inhibitors tested were the targeted therapies fingolimod (1 µM) plus gefitinib (1 µM), doxorubicin (10 nM), or the targeted therapies plus doxorubicin. Plates were transferred to the IncuCyte live-cell imager (Essen BioScience, Ann Arbor, MI, USA), and incubated for 120 h, with images collected every 3 h.

### Murine Models of Human TNBC

All animal procedures were approved by the institutional Animal Ethics Committee (Protocols RESP/14/280 and RESP/15/103), and followed recently described protocols (5). In brief, xenograft tumors were grown from human basal-like TNBC cell lines, implanted into the fourth left mammary fat pad of 8-week-old female BALB/c nude (immune-deficient) mice. For both MDA-MB-468 and HCC1806 tumors, 5 × 10^6^ cells were implanted in a volume of 150 µL, which included 50 µL of Matrigel (BD Biosciences, Franklin Lakes, NJ, USA). Tumors were allowed to develop until they reached a volume of 100 mm^3^, as determined by Vernier caliper measurement. For each cell type, randomized groups of tumor-bearing mice were then started on 3-times weekly i.p. injections of a combination of fingolimod (5 mg/Kg) plus gefitinib (25 mg/Kg), or vehicle, for up to 12 weeks. We previously showed that this drug combination substantially increased survival time in mice bearing both of these tumor types (5). For both tumor models, some studies included two additional groups of mice bearing ~100 mm^3^ tumors, treated with doxorubicin, 2 mg/Kg i.p., once weekly for 6 weeks, either alone or together with the fingolimod-gefitinib combination as described above (3-times weekly for up to 12 weeks). A higher doxorubicin dose (4 mg/Kg), or administration for more than 6 weeks, both caused toxicity (weight loss and/or death) that exceeded our ethical guidelines. Each mouse was terminated when its tumor reached the ethically approved endpoint of 1,000 mm^3^, otherwise 12 weeks after cell implantation.

### Immunohistochemistry (IHC) and Western Blotting

Immunohistochemistry staining for IGFBP-3, the proliferation marker Ki67, and the apoptosis marker cleaved caspase-3 (CCasp-3), was carried out as described previously (5). In brief, 4 µm sections of formalin-fixed paraffin-embedded tumor samples were incubated with antibodies against Ki67 (ab66155, 1:600, Abcam, Melbourne, VIC, Australia), CCasp-3 (Asp175) (#9661, 1:200, Cell Signaling), IGFBP-3 (in-house antiserum R-100, 1:2,000), and isotyped-matched IgG antibodies or rabbit serum alone, with nuclear counterstaining using Mayer’s hematoxylin and Scott’s bluing solution. For all three markers, the percentage of positive cells was scored for each tumor following recommendations for Ki67 analysis (19), calculated from the five highest staining areas at 10× magnification for each slide. Nuclear IGFBP-3 immunoreactivity was detected by western blotting of solubilized nuclei isolated from cell cultures, after immunoprecipitation using R-100 anti-IGFBP-3 Fab fragment conjugated to agarose beads, as previously described (20).

### Statistical Analysis

Kaplan–Meier survival analyses were undertaken using SPSS v.22 for Mac (IBM Corp., Armonk, New York, NY, USA). IHC scores were coded as low (≤the median value) or high (>the median value). Mice with MDA-MB-468 tumors below the ethical endpoint of 1,000 mm^3^ at day 82 after cell implantation were regarded as survivors. For HCC1806 tumors, all mice had reached the ethical endpoint before 12 weeks (i.e., there were no survivors). Linear and non-linear (exponential) correlations between IHC scores were fitted using Deltagraph v.7 (Red Rock Software, Salt Lake City, UT, USA) with *P* values calculated by SPSS.

## Results

### Nuclear IGFBP-3 Is Associated With Poor Outcome in TNBC Xenografts

**Figure 1:**
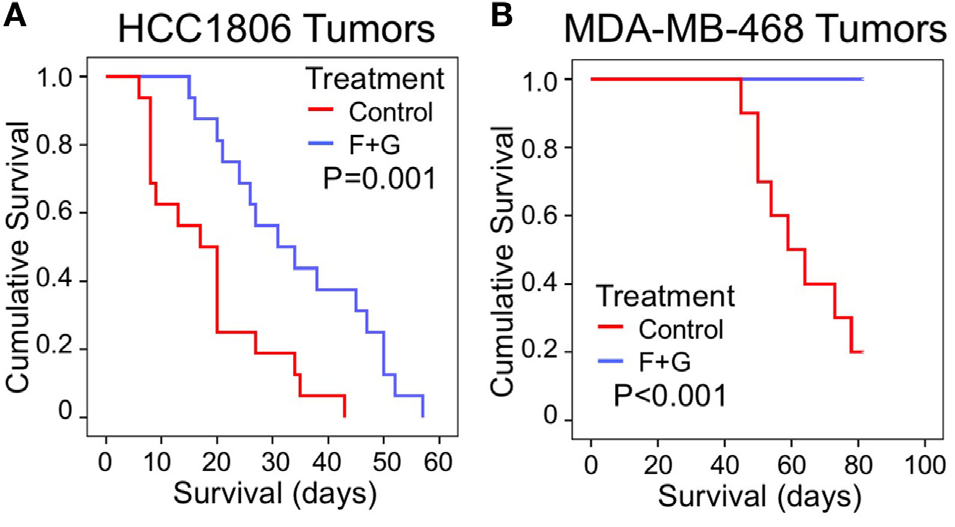
The effect of combination (fingolimod + gefitinib) treatment on the survival of nude mice bearing human xenograft TNBC tumors. **(A,B)** Kaplan–Meier survival curves compare the effect of control (no treatment) or combination fingolimod + gefitinib (F + G) therapy on survival of mice bearing HCC1806 **(A)** or MDA-MB-468 **(B)** xenograft tumors. For ethical reasons mouse survival is defined as tumor size below 1,000 mm^3^. For HCC1806, control: *n* = 16; combination: *n* = 16. For MDA-MB-468, control: *n* = 10; combination: *n* = 10.

As recently reported (5), the combination of SphK inhibition with fingolimod and EGFR kinase inhibition with gefitinib (F + G) was significantly inhibitory to the proliferation of both HCC1806 and MDA-MB-468 basal-like TNBC tumors. For HCC1806 tumors, mean mouse survival (measured for ethical reasons as the time for tumors to reach 1,000 mm^3^) was increased 87% by treatment compared to untreated controls, from 18.5 ± 2.8 to 34.6 ± 3.5 days (mean ± SEM, *P* = 0.001), while for MDA-MB-468 tumors, no tumors in treated mice had reached 1,000 mm^3^ after 82 days, compared to 8/10 tumors in control mice (*P* < 0.001). Figures 1A,B shows Kaplan–Meier survival curves for combination-treated vs. control-treated mice for HCC1806 and MDA-MB-468 tumors, respectively.

**Figure 2:**
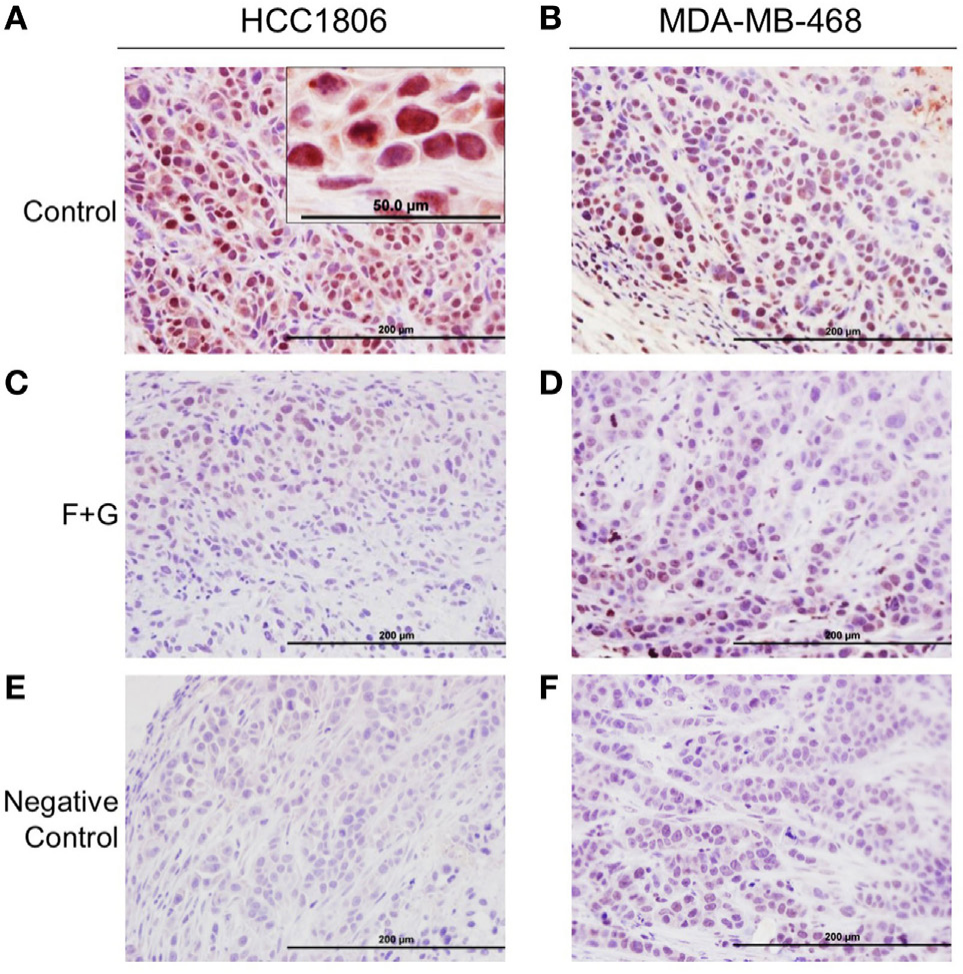
Insulin-like growth factor binding protein-3 (IGFBP-3) immunohistochemistry of HCC1806 and MDA-MB-468 tumors. **(A,B)** Representative IGFBP-3 staining of each tumor type from control mice, showing strong nuclear localization. **(C,D)** Representative IGFBP-3 staining of each tumor type from mice treated with fingolimod + gefitinib (F + G). **(E,F)** Negative control staining—non-immune rabbit serum at the same dilution as IGFBP-3 antiserum. Bar = 200 µm. Inset: higher magnification of HCC1806 section from control tumor. Bar = 50 µm.

To evaluate the relationship between tumor IGFBP-3 immunohistochemical staining and mouse survival, tumor tissues were stained for IGFBP-3 as well as the proliferation marker, Ki67 and the apoptosis marker, CCasp-3. Representative examples of staining patterns have been reported previously (5). Notably, IGFBP-3 staining, using a high-titer, primate-specific antiserum that does not detect murine IGFBP-3, was predominantly nuclear in both tumor types, as shown in Figures 2A,B. For both cell types, nuclear IGFBP-3 IHC staining in tumor sections was greatly decreased in combination-treated mice compared to controls (Figures 2C,D). Figures 2E,F shows negative staining controls for both tumor types.

**Figure 3:**
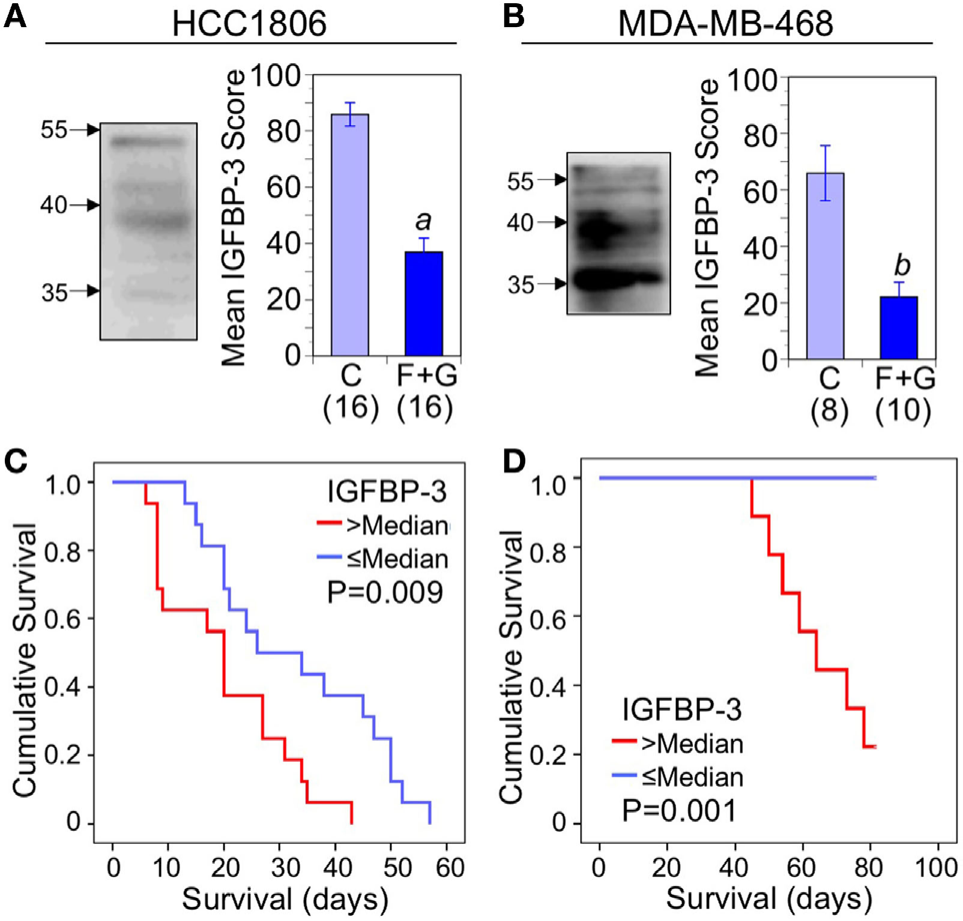
The relationship between tumor nuclear insulin-like growth factor binding protein-3 (IGFBP-3) and mouse survival. **(A,B)** Left: western blot of immunoreactive IGFBP-3 after immunoprecipitation from solubilized nuclei isolated from cultured cells; molecular weight markers in kDa are indicated by arrows. Right: quantitation of immunohistochemistry (IHC) scores for nuclear IGFBP-3 in control mice and mice treated with fingolimod + gefitinib (F + G). For HCC1806 **(A)** and MDA-MB-468 **(B)** tumors, mean scores ± SEM are shown, numbers of mice in parentheses. Comparison with controls (2-sided *t*-test): (*a*) *P* < 0.001; (*b*) *P* = 0.001. **(C,D)** Kaplan–Meier survival curves compare the effect of tumor nuclear IGFBP-3 IHC scores > or ≤ the median value on survival of mice bearing HCC1806 **(C)** or MDA-MB-468 **(D)** xenograft tumors. For HCC1806, *n* (>median) = 16; *n* (≤median) = 16. For MDA-MB-468, *n* (>median) = 9; *n* (≤median) = 9.

Nuclear IGFBP-3, examined by western blotting of nuclear extracts from both HCC1806 and MDA-MB-468 cell lines, typically showed diffuse bands of approximately 40 kDa as well as immunoreactivity around 35 kDa which may represent partially proteolyzed or underglycosylated IGFBP-3. The origin of diffuse IGFBP-3 immunoreactivity around 55 kDa is unknown (Figures 3A,B; left panels). Figures 3A,B (right panels) show mean IHC scores for IGFBP-3 from control and combination-treated mice. For both HCC1806 (A) and MDA-MB-468 (B) tumors, IGFBP-3 staining was significantly decreased. Kaplan–Meier survival analysis indicated a strong association between nuclear IGFBP-3 and mouse survival, IGFBP-3 IHC scores above the median value being associated with poor survival for both HCC1806 tumors (*P* = 0.009; Figure 3C) and MDA-MB-468 tumors (*P* = 0.001; Figure 3D).

**Figure 4:**
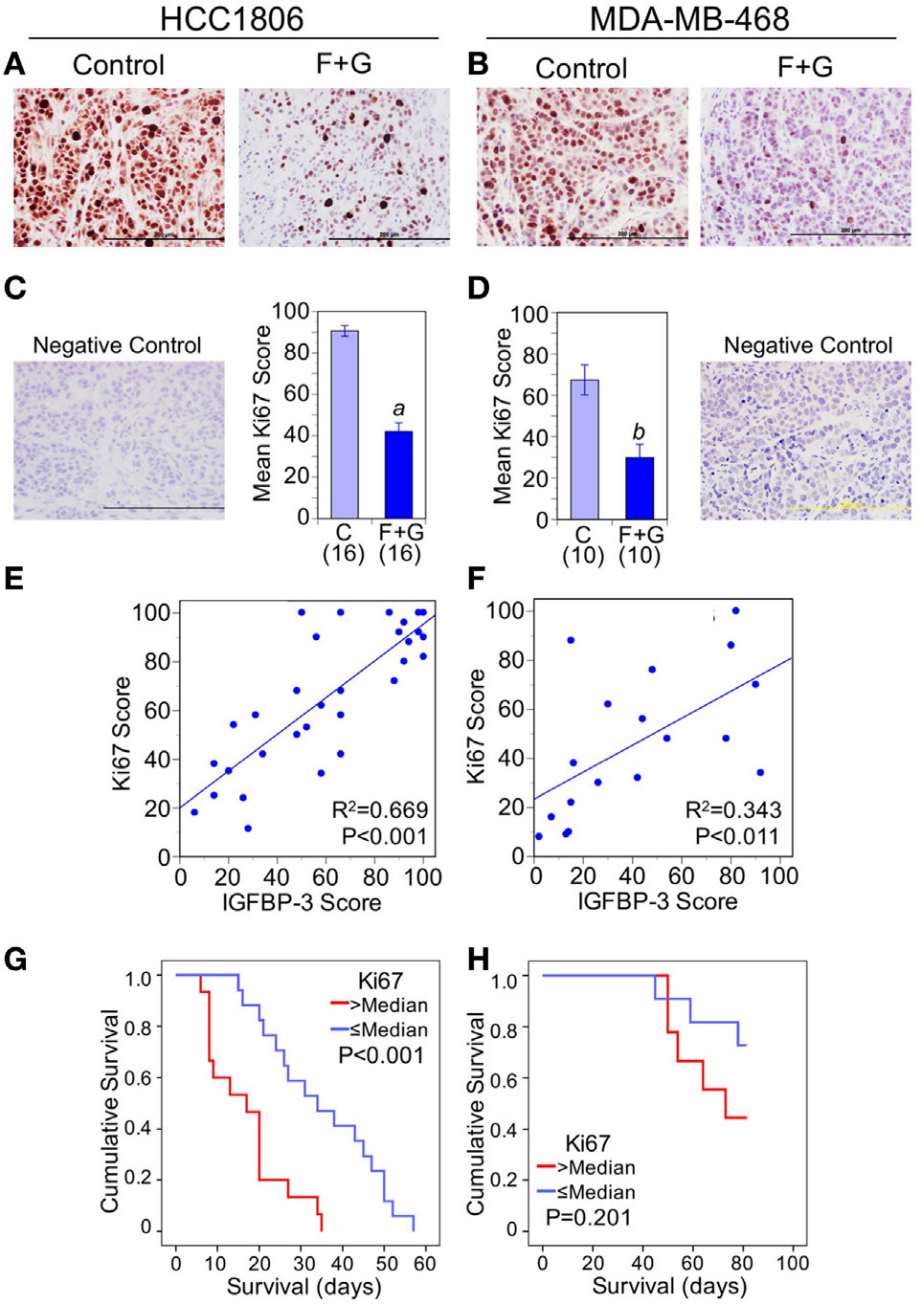
The relationship between tumor Ki67 and mouse survival. **(A,B)** Representative Ki67 staining of each tumor type from control mice and mice treated with fingolimod + gefitinib (F + G). **(C,D)** Quantitation of immunohistochemistry (IHC) scores for Ki67 in control and combination-treated mice. For HCC1806 **(C)** and MDA-MB-468 **(D)** tumors, mean scores ± SEM are shown, numbers of mice in parentheses. Comparison with controls (2-sided *t*-test): (*a*) *P* < 0.001; (*b*) *P* = 0.001. Also shown are IgG isotype negative staining controls for both tumor types. **(E,F)** Nuclear insulin-like growth factor binding protein-3 scores are positively correlated with Ki67 scores for both HCC1806 **(E)** and MDA-MB-468 **(F)** tumors. Linear regression lines are shown with *R*^2^ and *P* values indicated. **(G,H)** Kaplan–Meier survival curves compare the effect of tumor Ki67 IHC scores > or ≤ the median value on survival of mice bearing HCC1806 **(G)** or MDA-MB-468 **(H)** xenograft tumors. For ethical reasons mouse survival is defined as tumor size below 1,000 mm^3^. For HCC1806, *n* (>median) = 15; *n* (≤median) = 17. For MDA-MB-468, *n* (>median) = 9; *n* (≤median) = 11.

To assess whether decreased nuclear IGFBP-3 was linked to changes in proliferation or apoptosis, we examined the effect of combination F + G treatment on Ki67 and CCasp-3. As previously reported (5), the combination treatment significantly decreased cell proliferation, as indicated by Ki67 staining (Figures 4A–D). Nuclear IGFBP-3 staining was positively correlated with nuclear Ki67, more strongly in HCC1806 tumors (Figure 4E) than MDA-MB-468 tumors (Figure 4F). This was reflected in the Kaplan–Meier survival curves, in which high Ki67 levels (above the median) were strongly associated with poor survival for HCC1806 tumors (Figure 4G), but not with MDA-MB-468 tumors (Figure 4H).

**Figure 5:**
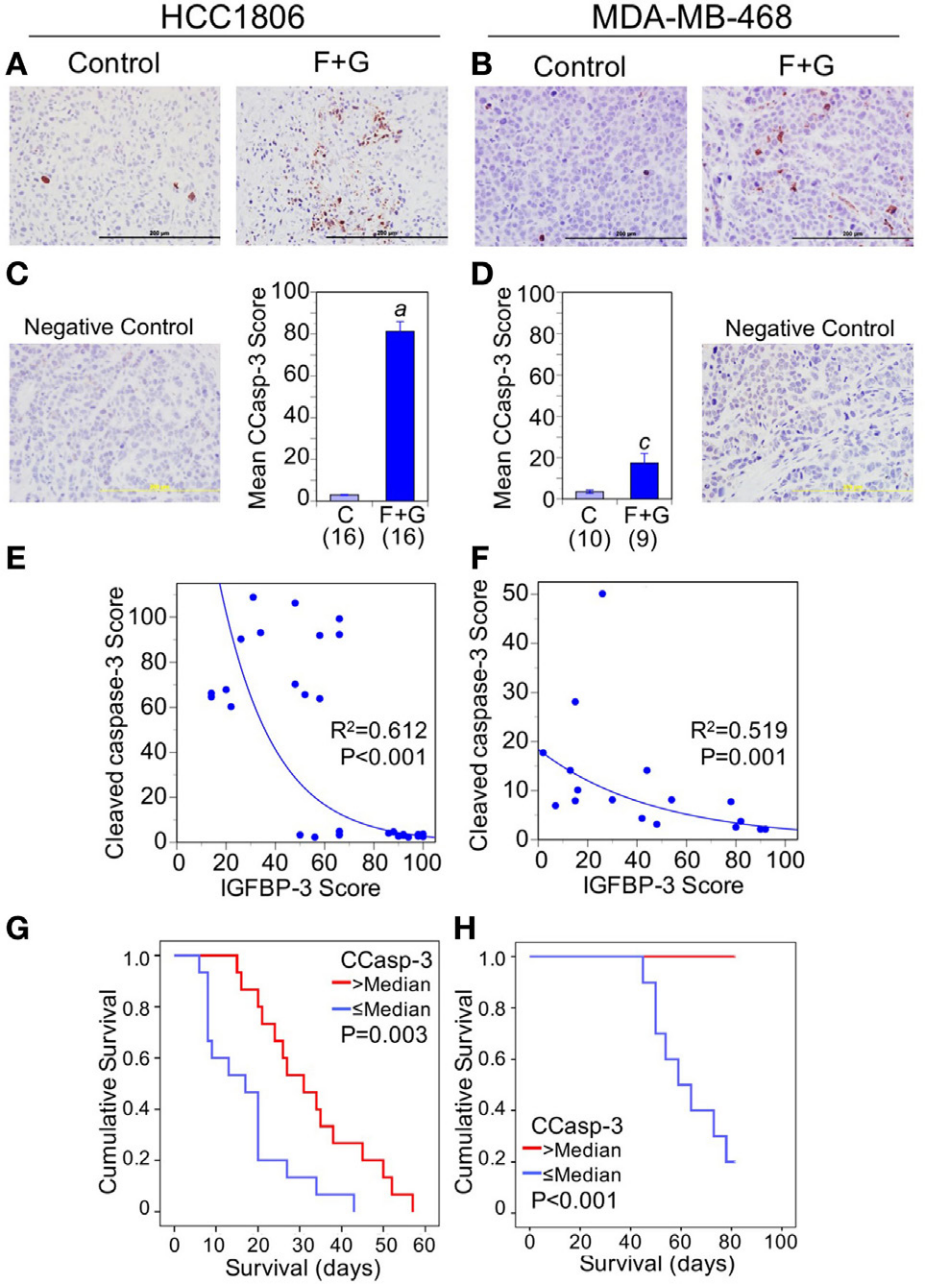
The relationship between tumor cleaved caspase-3 (CCasp-3) and mouse survival. **(A,B)** Representative CCasp-3 staining of each tumor type from control mice and mice treated with fingolimod + gefitinib (F + G). **(C,D)** Quantitation of immunohistochemistry (IHC) scores for CCasp-3 in control and combination-treated mice. For HCC1806 **(C)** and MDA-MB-468 **(D)** tumors, mean scores ± SEM are shown, numbers of mice in parentheses. Comparison with controls (2-sided *t*-test): (*a*) *P* < 0.001; (*c*) *P* = 0.006. Also shown are IgG isotype negative staining controls for both tumor types. **(E,F)** Nuclear insulin-like growth factor binding protein-3 scores are negatively correlated with CCasp-3 scores for both HCC1806 **(E)** and MDA-MB-468 **(F)** tumors. Exponential regression lines are shown with *R*^2^ and *P* values indicated. **(G,H)** Kaplan–Meier survival curves compare the effect of tumor CCasp-3 IHC scores > or ≤ the median value on survival of mice bearing HCC1806 **(G)** or MDA-MB-468 **(H)** xenograft tumors. For ethical reasons mouse survival is defined as tumor size below 1,000 mm^3^. For HCC1806, *n* (>median) = 15; *n* (≤median) = 15. For MDA-MB-468, *n* (>median) = 9; *n* (≤median) = 10.

Apoptosis, as indicated by caspase-3 cleavage, was also strongly induced by combination F + G treatment (Figures 5A–D). The increased apoptosis was particularly notable in HCC1806 tumors, in which CCasp-3 staining increased over 25-fold. Nuclear IGFBP-3 was inversely correlated with CCasp-3 staining for both tumor types (Figures 5E,F), and survival analysis showed that CCasp-3 staining above the median level was significantly associated with improved mouse survival (Figures 5G,H). Collectively these data suggest that, in these xenograft models of human basal-like TNBC, a high nuclear IGFBP-3 level may be a poor prognostic feature, associated with high tumor proliferation and decreased apoptosis.

### Comparison Between Combination Targeted Therapy and Chemotherapy

**Figure 6:**
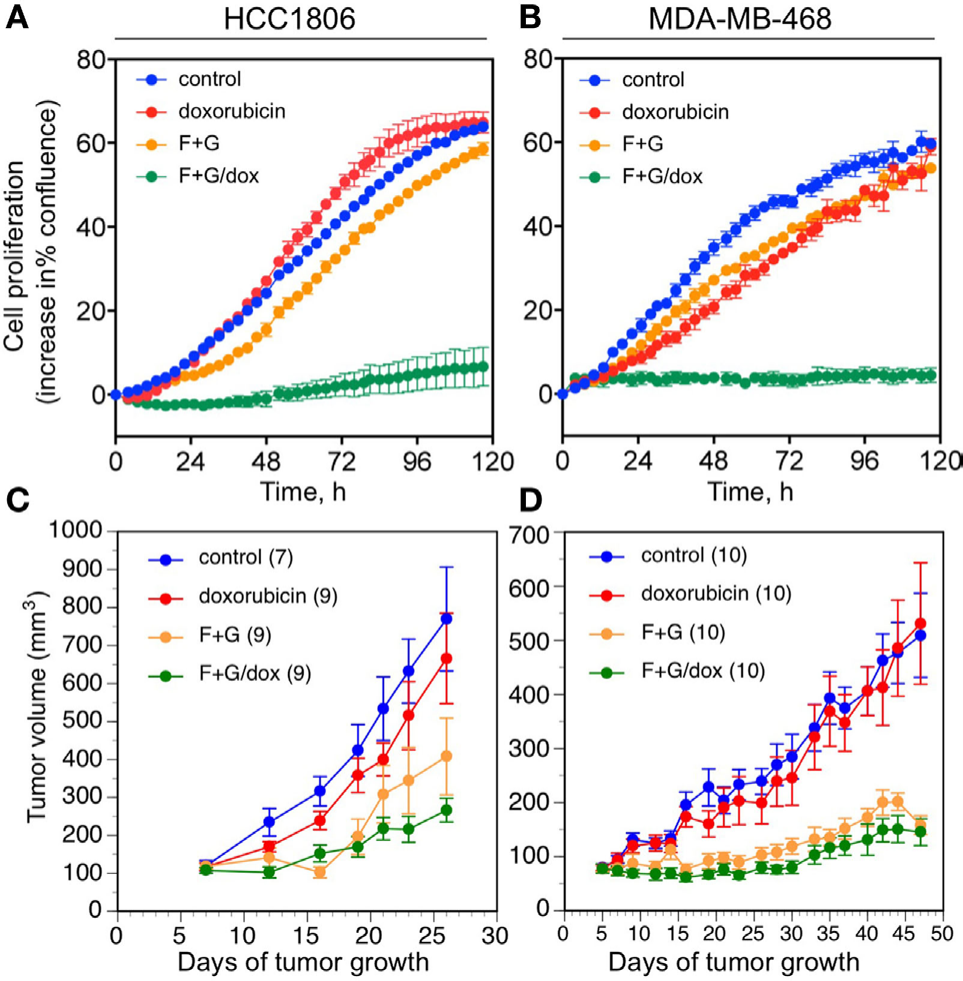
The effect of kinase inhibitors and doxorubicin on the proliferation of basal-like triple-negative breast cancer cells. **(A,B)** Cell culture studies using the IncuCyte live-cell imager. **(A)** HCC1806 cells; **(B)** MDA-MB-468 cells. Data are expressed as the change in percent confluence, corrected for confluence at time zero; mean values ± SEM at each time point, from triplicate wells for each treatment. Similar results were obtained in three replicate experiments for each cell line. Treatments are: control (blue); doxorubicin, 10 nM (red); fingolimod, 1 µM + gefitinib, 1 µM (F + G; orange); and F + G plus doxorubicin (green). **(C,D)** *In vivo* studies of orthotopic xenograft tumor growth in nude mice; mean values ± SEM, numbers in parentheses. **(C)** HCC1806 tumors. Repeated measures ANOVA up to day 26: overall effect of treatment, *P* = 0.003. *Post hoc* Tukey’s test: F + G vs. control, *P* = 0.036; F + G/dox vs. control, *P* = 0.004; F + G/dox vs. dox, *P* = 0.045. **(D)** MDA-MB-468 tumors. Repeated measures ANOVA up to day 47: overall effect of treatment, *P* < 0.001. *Post hoc* Tukey’s test: F + G vs. control, *P* = 0.001; F + G/dox vs. control, *P* < 0.001; F + G/dox vs. dox, *P* = 0.001. Treatments are: control (blue); doxorubicin, 2 mg/Kg (red); fingolimod, 5 mg/Kg + gefitinib, 25 mg/Kg (F + G; orange); and F + G plus doxorubicin (green). See Section “Materials and Methods” for further details.

In the absence of any approved targeted therapies for TNBC, cytotoxic chemotherapy is regarded as the front-line treatment (6). We, therefore, evaluated the efficacy of F + G therapy in combination with the widely used anthracycline chemotherapy drug, doxorubicin. We previously established concentrations of the two kinase inhibitors that showed a strongly synergistic cytostatic effect *in vitro* in various TNBC cell lines (5). In this study, their concentrations were lowered to ensure that the drug combination, when used alone, would have minimal cytostatic effect, in order to examine the additional effect of doxorubicin. Figures 6A,B shows that 1 µM fingolimod + 1 µM gefitinib had little cytostatic effect, measured by IncuCyte real-time imaging over 120 h, in either HCC1806 or MDA-MB-468 cell cultures. Similarly, doxorubicin was minimally cytostatic at 10 nM. However, treatment with doxorubicin plus the targeted drug combination resulted in almost complete inhibition of cell proliferation in both cell lines, suggesting a synergistic effect between the kinase inhibitors with the chemotherapeutic agent (Figures 6A,B).

**Figure 7:**
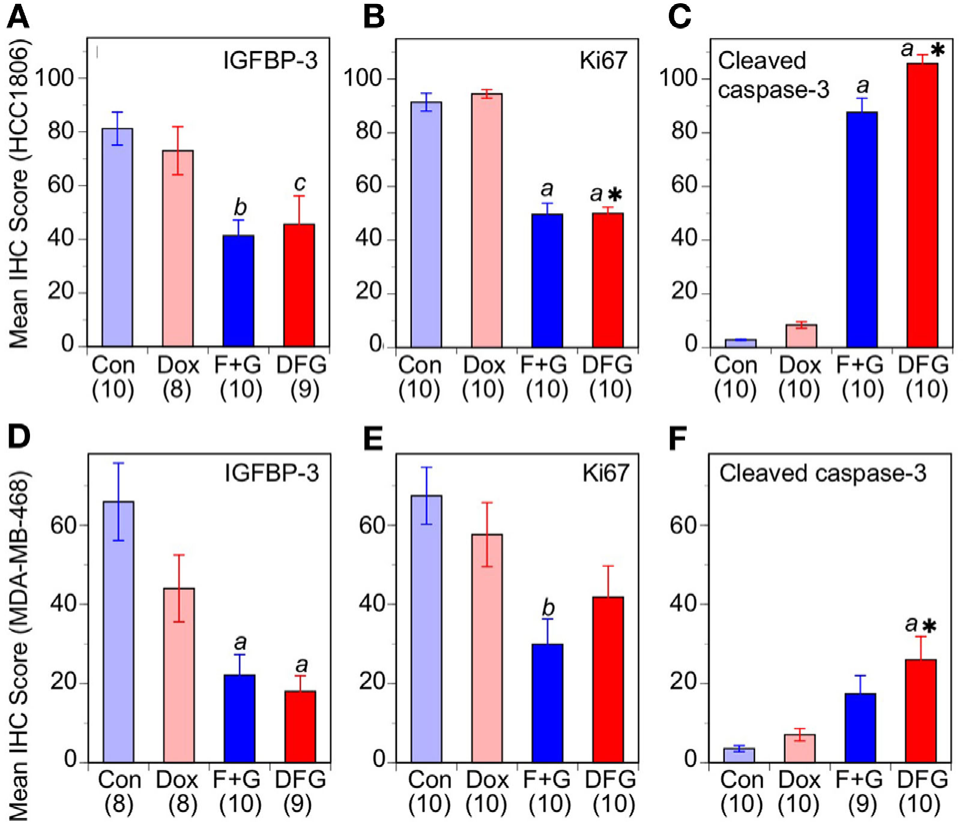
Response of basal-like triple-negative breast cancer tumors to doxorubicin and combination therapy. Immunohistochemistry scores for nuclear insulin-like growth factor binding protein-3, Ki67, and cleaved caspase-3 in mice with HCC1806 tumors **(A–C)** or MDA-MB-468 tumors **(D–F)** treated with vehicle alone (Con), doxorubicin (Dox), fingolimod + gefitinib (F + G), or Dox plus F + G (DFG). For both tumor types, data are mean values ± SEM, numbers in parentheses. Comparison with controls (one-way ANOVA, *post hoc* Tukey’s test): (*a*) *p* < 0.001; (*b*) *p* < 0.005; (*c*) *p* < 0.02. Comparison with Dox: *, *P* < 0.01.

To evaluate this effect *in vivo*, the two xenograft models of TNBC were treated with the combination F + G therapy plus doxorubicin. Doxorubicin was administered at the maximum tolerated dose (MTD) of 2 mg/Kg i.p. once weekly for 6 weeks, since a higher dose (4 mg/Kg) or longer duration resulted in drug-related toxicity in our BALB/c nude mouse model (data not shown). As shown in Figures 6C,D, doxorubicin at the highest tolerable dose of 2 mg/Kg had only a small inhibitory effect on the growth of either tumor type *in vivo*, not statistically significant, whereas the targeted combination was significantly inhibitory in both models as previously reported (5). Notably, and in contrast to the *in vitro* studies, treatment with both doxorubicin and the F + G combination did not significantly slow down the tumor growth rate in either model, beyond the effect seen with F + G alone. Immunohistochemical analysis of tumors for IGFBP-3, Ki67, and CCasp-3 showed that, at the doses tested in these experiments, doxorubicin had no significant effect either administered alone or in combination with F + G treatment, in either HCC1806 (Figures 7A–C) or MDA-MB-468 (Figures 7D–F) tumors.

## Discussion

### IGFBP-3 As a Therapeutic Target and Biomarker

The first goal of this study was to evaluate tumor IGFBP-3 abundance for potential value as a prognostic indicator in basal-like triple-negative breast tumors, using as model systems, two basal-like TNBC cell lines grown as orthotopic xenograft tumors in nude mice. Our studies over several years have shown that IGFBP-3 stimulates an oncogenic pathway in which the activation of SphK 1, leading to increased sphingosine-1-phosphate generation, results in the transactivation of EGFR which drives tumor proliferation (12, 13). The importance of SphK/sphingosine-1-phosphate in oncogenesis, and its potential as a therapeutic target in breast and other cancers, has been studied extensively (21, 22), and a recent study in a metastatic TNBC cell model (23) has confirmed our earlier observations that SphK inhibition decreases EGFR-dependent cell proliferation and survival.

Although IGFBP-3 itself, which is abundant in the circulation (14) and in ER-negative breast cancer tissue (24), is a challenging therapeutic target, we showed that IGFBP-3-dependent oncogenic signaling can be successfully targeted by dual therapy with an EGFR kinase inhibitor and a SphK inhibitor, giving a highly synergistic inhibitory effect (5, 13). This may offer benefits over EGFR-directed therapies used alone, since both EGFR kinase inhibitors and monoclonal antibodies have failed to demonstrate consistent results in the treatment of TNBC (25). Since we found, using cell models, that IGFBP-3 downregulation attenuated the synergism between the EGFR and SphK inhibitors, we proposed that tumor IGFBP-3 levels might act as a biomarker for the efficacy of the combination therapy (5).

As a key regulator of the bioavailability of circulating IGF-1 and IGF-2, IGFBP-3 has major effects outside the cell (14), but its intracellular (including intranuclear) actions are also well documented (15, 26). IGFBP-3 has been identified in the cell nucleus in a variety of healthy and cancerous human tissues, including non-malignant colon (27), lung (28), articular cartilage (29), and bone (30), as well as malignant colon (27), liver (31), Barrett’s tissue associated with esophageal cancer (32), and prostate (18) [including reactive stroma (33)] tissue.

### Nuclear and Cytoplasmic IGFBP-3 in Cancer

Although *IGFBP3* is epigenetically suppressed in some cancers, with an apparent tumor suppressor role (34, 35), in other cancer types it shows high expression in association with high tumor grade and/or poor patient survival, although often the IGFBP-3 protein is located predominantly in the cytoplasm. For example, IGFBP-3 is highly overexpressed in the cytoplasm of high-grade clear cell renal cell carcinomas compared to low-grade or normal kidney (36). A similar finding was reported in brain tumors, with highest IGFBP-3 levels by both gene expression and IHC found in glioblastoma (grade IV) compared to normal brain and astrocytomas of lower grade (37). High IGFBP-3 staining in glioblastoma patients was significantly associated with shorter survival, and was described as “mostly confined to the cytoplasm” although some positive nuclei were also evident (37). In pancreatic endocrine (i.e., islet cell) neoplasms (38) and melanoma (39), high tumor IGFBP-3 levels by IHC are associated with increased metastasis, but again the staining is predominantly cytoplasmic. In head and neck squamous cell carcinoma, high IGFBP-3 staining was associated with higher clinical stage (40) and decreased time to progression (41), and was similarly mainly seen in the cytoplasm. In an IHC study of breast cancer tissue sections, in which high IGFBP-3 staining showed a trend (non-significant) of association with worse patient survival, “no clear evidence” of nuclear IGFBP-3 staining was reported (42).

In contrast, in a study of hepatocellular carcinoma Yan et al. (31) scored nuclear IGFBP-3 staining, finding an association between *low* nuclear IGFBP-3 and several markers of poor prognosis. Finally, Seligson et al. (18) observed increased IGFBP-3 staining in both the cytoplasm and nucleus of prostate cancers compared to benign tissue, but only the nuclear staining was significantly prognostic for cancer recurrence, being more highly predictive than baseline PSA or any other pathological marker. Therefore, both the subcellular localization and prognostic value of tumor IGFBP-3 staining appears to be highly dependent on tumor type.

We recently reported that human basal-like TNBC cells growing as xenograft tumors showed predominantly nuclear IGFBP-3 staining (5). We have now extended this observation by showing in two xenograft models that high levels of nuclear IGFBP-3 (i.e., above the median value), measured by IHC, are significantly associated with shorter mouse survival time. Mature human IGFBP-3 is a glycoprotein of 264 amino acids with a core molecular weight of 28.7 kDa, increased to approximately 40 kDa by *N*-glycosylation on residues 89, 109, and 172. Variable glycosylation leads to the appearance of diffuse bands around 40 kDa when analyzed by SDS-PAGE (43–45). The IGFBP-3 structure may be viewed as three domains of approximately equal size: cysteine-rich amino- and carboxyterminal domains that bind IGF-1 and IGF-2 cooperatively (46), and a central or linker domain that is the major site for posttranslational modification. Limited proteolysis in the central domain may be involved in the release of IGFs from the high-affinity binding pocket, and accounts for frequently observed IGFBP-3 fragments in the circulation and tissues (26, 45).

Although nuclei were not isolated from xenograft tumors in this study, nuclear extracts of the corresponding cell lines showed IGFBP-3 immunoreactive bands of approximately 40 kDa that may represent glycosylated isoforms of the full-length protein, as well as larger (~55 kDa) and smaller (~35 kDa) bands that may represent glycosylation and/or proteolysis variants. Our studies do not indicate which of these bands are downregulated by drug treatment, or whether the size distribution of the variants changes on treatment. Nuclear IGFBP-3 IHC staining correlated positively with nuclear Ki67 and inversely with CCasp-3, consistent with the role of IGFBP-3 in promoting proliferation and survival in basal-like TNBC cells. If extrapolated to TNBC tumors in patients, our findings suggest that a high nuclear IGFBP-3 score by IHC, complementing a high Ki67 score and a low CCasp-3 score, may be prognostic for poor patient survival in women with TNBC. Further refinement of such a prognostic test might be possible with more detailed analysis of the structure and size distribution of IGFBP-3 variants in tumors. Since treatment with combination fingolimod and gefitinib significantly prolonged survival in our mouse models, this combination therapy might be considered for evaluation in clinical trials.

### Effect of Doxorubicin in TNBC Models

Anthracycline drugs (e.g., doxorubicin and epirubicin) are a standard component of adjuvant chemotherapy for women with TNBC (47). The second goal of this study was to evaluate whether the SphK/EGFR inhibitor combination (F + G) might act cooperatively with doxorubicin as an effective treatment for TNBC. Since doxorubicin has well-documented toxicity toward the brain, liver, kidney, and notably the heart (48), lowering the dose by the addition of a targeted therapy might be clinically advantageous. In this pre-clinical study, the dose of doxorubicin, 2 mg/Kg i.p. weekly for 6 weeks was used to avoid toxicity seen at higher or more prolonged dosing schedules in the BALB/c nude mice. In the initial cell growth studies *in vitro*, a strong combination effect between F + G and doxorubicin was observed, in which sub-cytostatic doses of each caused almost complete cytostatsis when combined. However, this trend was not recapitulated in two xenograft tumor models in which doxorubicin, administered alone or in combination with the kinase inhibitors, showed no significant effect on tumor volume or on cellular markers of proliferation and apoptosis. Similarly, IGFBP-3 staining did not show a differential response to doxorubicin in our models. Although *IGFBP3* is activated by wild-type p53 (49), and, therefore, expected to be induced by chemotherapy, we have previously shown that in some TNBC cell lines with gain-of-function p53 mutations, IGFBP-3 is paradoxically downregulated by DNA-damaging chemotherapeutic drugs (50). Effective doxorubicin activity might, therefore, be expected to downregulate IGFBP-3 (as seen for the effective combination kinase inhibitor therapy), but this was not observed in our study.

While it is unclear why the *in vivo* experiments failed to show any significant beneficial effect of doxorubicin in these models, a likely limiting factor was the MTD of 2 mg/Kg i.p. weekly for 6 weeks in our BALB/c nude mouse model. A similar doxorubicin dose for 3 weeks gave only a “minimum to partial response” in patient-derived TNBC xenograft tumors in NOD-SCID mice (51), whereas 1.5 mg/Kg weekly as an i.v. bolus significantly reduced the volume of MDA-MB-231 TNBC xenograft tumors in nude mice (52). In our study, the very small and non-significant effect of doxorubicin provided an opportunity to observe a cooperative inhibitory effect with F + G treatment, but no such effect was observed in the tumor models used. It is possible that by increasing doxorubicin tolerability through the use of liposomes or other nano-delivery systems (48), a tolerable dose may have been achieved at which doxorubicin would complement the effects of the kinase inhibitors. However, our study reinforces the conclusion (5) that, since both fingolimod (53) and gefitinib (54) are FDA-approved drugs (though not for breast cancer), inhibiting IGFBP-3-dependent oncogenic signaling by F + G therapy is a plausible approach to targeting basal-like TNBC tumors.

## Conclusion

Insulin-like growth factor binding protein-3-dependent signaling may be viewed as a controversial target for cancer therapy, since in some cancers *IGFBP3* appears to act as a tumor suppressor gene, but our data suggest the plausibility of this approach for women with TNBC. IGFBP-3 is highly expressed and a poor prognostic feature in ER-negative breast cancers, and initiates an oncogenic signaling cascade in both *in vitro* and *in vivo* pre-clinical models of basal-like TNBC. Combined EGFR and SphK inhibition, to block IGFBP-3-dependent signaling, synergistically inhibits cell proliferation and this effect is attenuated by IGFBP-3 downregulation (5). This pre-clinical study has shown that combination of fingolimod + gefitinib-targeted therapy is more effective than doxorubicin at its MTD, in inhibiting the growth of basal-like TNBC xenograft tumors. IGFBP-3, detected predominantly in the cell nucleus in these tumors, is less abundant in more slowly growing tumors, and its high nuclear staining is similar to high Ki67 staining as a marker of decreased mouse survival. We, therefore, conclude that fingolimod + gefitinib combination therapy for basal-like TNBC tumors with high IGFBP-3 might be suitable for evaluation in a neoadjuvant setting as an effective alternative to cytotoxic chemotherapy.

## Ethics Statement

This study was carried out in accordance with the recommendations of, and with the approval of, the Northern Sydney Local Health District Animal Ethics Committee (Protocols RESP/14/280 and RESP/15/103).

## Author Contributions

All authors contributed to the conception and design of the study. SJ and JM contributed to the acquisition, analysis, and interpretation of data. RB contributed to data analysis and interpretation and wrote the manuscript. All authors contributed to its critical revision and approved the final version for publication.

## Conflict of Interest Statement

All authors confirm that they have no potential or actual conflicts of interest with regards to this work. The handling editor and reviewer AH declared their involvement as co-editors in the research topic, and confirm the absence of any other collaboration.

## References

[1] Reis-Filho JS Pusztai L Gene expression profiling in breast cancer: classification, prognostication, and prediction. Lancet (2011) 378:1812–23. 10.1016/S0140-6736(11)61539-0 22098854

[2] Russnes HG Lingjaerde OC Borresen-Dale AL Caldas C. Breast cancer molecular stratification: from intrinsic subtypes to integrative clusters. Am J Pathol (2017) 187:2152–62. 10.1016/j.ajpath.2017.04.022 28733194

[3] Lehmann BD Bauer JA Chen X Sanders ME Chakravarthy AB Shyr Y Identification of human triple-negative breast cancer subtypes and preclinical models for selection of targeted therapies. J Clin Invest (2011) 121:2750–67. 10.1172/JCI45014 21633166 PMC3127435

[4] Penault-Llorca F Viale G. Pathological and molecular diagnosis of triple-negative breast cancer: a clinical perspective. Ann Oncol (2012) 23(Suppl 6):vi19–22. 10.1093/annonc/mds190 23012297

[5] Martin JL Julovi SM Lin MZ de Silva HC Boyle FM Baxter RC. Inhibition of basal-like breast cancer growth by FTY720 in combination with epidermal growth factor receptor kinase blockade. Breast Cancer Res (2017) 19:90. 10.1186/s13058-017-0882-x 28778177 PMC5545026

[6] Bianchini G Balko JM Mayer IA Sanders ME Gianni L. Triple-negative breast cancer: challenges and opportunities of a heterogeneous disease. Nat Rev Clin Oncol (2016) 13:674–90. 10.1038/nrclinonc.2016.66 27184417 PMC5461122

[7] Emens LA. Breast cancer immunotherapy: facts and hopes. Clin Cancer Res (2018) 24:511–20. 10.1158/1078-0432.CCR-16-3001 28801472 PMC5796849

[8] Scully T Scott CD Firth SM Sedger LM Pintar JE Twigg SM Enhancement of mammary tumour growth by IGFBP-3 involves impaired T cell accumulation. Endocr Relat Cancer (2018) 25:111–22. 10.1530/ERC-17-0384 29217518

[9] Probst-Hensch NM Steiner JH Schraml P Varga Z Zurrer-Hardi U Storz M IGFBP2 and IGFBP3 protein expressions in human breast cancer: association with hormonal factors and obesity. Clin Cancer Res (2010) 16:1025–32. 10.1158/1078-0432.CCR-09-0957 20103684

[10] Yu H Levesque MA Khosravi MJ Papanastasiou-Diamandi A Clark GM Diamandis EP Associations between insulin-like growth factors and their binding proteins and other prognostic indicators in breast cancer. Br J Cancer (1996) 74:1242–7. 10.1038/bjc.1996.523 8883411 PMC2075943

[11] Rocha RL Hilsenbeck SG Jackson JG VanDenBerg CL Weng C Lee AV Insulin-like growth factor binding protein-3 and insulin receptor substrate-1 in breast cancer: correlation with clinical parameters and disease-free survival. Clin Cancer Res (1997) 3:103–9. 9815544

[12] Martin JL Lin MZ McGowan EM Baxter RC. Potentiation of growth factor signaling by insulin-like growth factor-binding protein-3 in breast epithelial cells requires sphingosine kinase activity. J Biol Chem (2009) 284:25542–52. 10.1074/jbc.M109.007120 19633297 PMC2757955

[13] Martin JL de Silva HC Lin MZ Scott CD Baxter RC. Inhibition of insulin-like growth factor-binding protein-3 signaling through sphingosine kinase-1 sensitizes triple-negative breast cancer cells to EGF receptor blockade. Mol Cancer Ther (2014) 13:316–28. 10.1158/1535-7163.MCT-13-0367 24337110

[14] Baxter RC. IGF binding proteins in cancer: mechanistic and clinical insights. Nat Rev Cancer (2014) 14:329–41. 10.1038/nrc3720 24722429

[15] Baxter RC. Nuclear actions of insulin-like growth factor binding protein-3. Gene (2015) 569:7–13. 10.1016/j.gene.2015.06.028 26074086 PMC4496269

[16] Liu B Lee HY Weinzimer SA Powell DR Clifford JL Kurie JM Direct functional interactions between insulin-like growth factor-binding protein-3 and retinoid X receptor-alpha regulate transcriptional signaling and apoptosis. J Biol Chem (2000) 275:33607–13. 10.1074/jbc.M002547200 10874028

[17] Cobb LJ Liu B Lee KW Cohen P. Phosphorylation by DNA-dependent protein kinase is critical for apoptosis induction by insulin-like growth factor binding protein-3. Cancer Res (2006) 66:10878–84. 10.1158/0008-5472.CAN-06-0585 17108124

[18] Seligson DB Yu H Tze S Said J Pantuck AJ Cohen P IGFBP-3 nuclear localization predicts human prostate cancer recurrence. Horm Cancer (2013) 4:12–23. 10.1007/s12672-012-0124-8 23011725 PMC3541441

[19] Dowsett M Nielsen TO A’Hern R Bartlett J Coombes RC Cuzick J Assessment of Ki67 in breast cancer: recommendations from the International Ki67 in breast cancer working group. J Natl Cancer Inst (2011) 103:1656–64. 10.1093/jnci/djr393 21960707 PMC3216967

[20] Lin MZ Marzec KA Martin JL Baxter RC. The role of insulin-like growth factor binding protein-3 in the breast cancer cell response to DNA-damaging agents. Oncogene (2014) 33:85–96. 10.1038/onc.2012.538 23178489

[21] Ogretmen B. Sphingolipid metabolism in cancer signalling and therapy. Nat Rev Cancer (2018) 18:33–50. 10.1038/nrc.2017.96 29147025 PMC5818153

[22] Geffken K Spiegel S Sphingosine kinase 1 in breast cancer. Adv Biol Regul (2017) 67:59–65. 10.1016/j.jbior.2017.10.005 29055687 PMC5807162

[23] Maiti A Takabe K Hait NC. Metastatic triple-negative breast cancer is dependent on SphKs/S1P signaling for growth and survival. Cell Signal (2017) 32:85–92. 10.1016/j.cellsig.2017.01.021 28108260 PMC5731460

[24] Figueroa JA Jackson JG McGuire WL Krywicki RF Yee D. Expression of insulin-like growth factor binding proteins in human breast cancer correlates with estrogen receptor status. J Cell Biochem (1993) 52:196–205. 10.1002/jcb.240520211 7690042

[25] Costa R Shah AN Santa-Maria CA Cruz MR Mahalingam D Carneiro BA Targeting epidermal growth factor receptor in triple negative breast cancer: new discoveries and practical insights for drug development. Cancer Treat Rev (2017) 53:111–9. 10.1016/j.ctrv.2016.12.010 28104566

[26] Firth SM Baxter RC. Cellular actions of the insulin-like growth factor binding proteins. Endocr Rev (2002) 23:824–54. 10.1210/er.2001-0033 12466191

[27] Miraki-Moud F Jenkins PJ Fairclough PD Jordan S Bustin SA Jones AM Increased levels of insulin-like growth factor binding protein-2 in sera and tumours from patients with colonic neoplasia with and without acromegaly. Clin Endocrinol (Oxf) (2001) 54:499–508. 10.1046/j.1365-2265.2001.01221.x 11318786

[28] Chang YS Kong G Sun S Liu D El-Naggar AK Khuri FR Clinical significance of insulin-like growth factor-binding protein-3 expression in stage I non-small cell lung cancer. Clin Cancer Res (2002) 8:3796–802. 12473592

[29] Hunziker EB Kapfinger E Martin J Buckwalter J Morales TI. Insulin-like growth factor (IGF)-binding protein-3 (IGFBP-3) is closely associated with the chondrocyte nucleus in human articular cartilage. Osteoarthritis Cartilage (2008) 16:185–94. 10.1016/j.joca.2007.06.008 17693100 PMC2364636

[30] Ressler S Radhi J Aigner T Loo C Zwerschke W Sergi C. Insulin-like growth factor-binding protein-3 in osteosarcomas and normal bone tissues. Anticancer Res (2009) 29:2579–87. 19596932

[31] Yan J Yang X Li L Liu P Wu H Liu Z Low expression levels of insulin-like growth factor binding protein-3 are correlated with poor prognosis for patients with hepatocellular carcinoma. Oncol Lett (2017) 13:3395–402. 10.3892/ol.2017.5934 28521445 PMC5431398

[32] Di Martino E Wild CP Rotimi O Darnton JS Olliver RJ Hardie LJ. IGFBP-3 and IGFBP-10 (CYR61) up-regulation during the development of Barrett’s oesophagus and associated oesophageal adenocarcinoma: potential biomarkers of disease risk. Biomarkers (2006) 11:547–61. 10.1080/13547500600896791 17056474

[33] Sampson N Zenzmaier C Heitz M Hermann M Plas E Schafer G Stromal insulin-like growth factor binding protein 3 (IGFBP3) is elevated in the diseased human prostate and promotes ex vivo fibroblast-to-myofibroblast differentiation. Endocrinology (2013) 154:2586–99. 10.1210/en.2012-2259 23720424

[34] Hanafusa T Yumoto Y Nouso K Nakatsukasa H Onishi T Fujikawa T Reduced expression of insulin-like growth factor binding protein-3 and its promoter hypermethylation in human hepatocellular carcinoma. Cancer Lett (2002) 176:149–58. 10.1016/S0304-3835(01)00736-4 11804742

[35] Torng PL Lin CW Chan MW Yang HW Huang SC Lin CT. Promoter methylation of IGFBP-3 and p53 expression in ovarian endometrioid carcinoma. Mol Cancer (2009) 8:120. 10.1186/1476-4598-8-120 20003326 PMC2799391

[36] Chuang ST Patton KT Schafernak KT Papavero V Lin F Baxter RC Over expression of insulin-like growth factor binding protein 3 in clear cell renal cell carcinoma. J Urol (2008) 179:445–9. 10.1016/j.juro.2007.09.106 18076934

[37] Santosh V Arivazhagan A Sreekanthreddy P Srinivasan H Thota B Srividya MR Grade-specific expression of insulin-like growth factor-binding proteins-2, -3, and -5 in astrocytomas: IGFBP-3 emerges as a strong predictor of survival in patients with newly diagnosed glioblastoma. Cancer Epidemiol Biomarkers Prev (2010) 19:1399–408. 10.1158/1055-9965.EPI-09-1213 20501753

[38] Hansel DE Rahman A House M Ashfaq R Berg K Yeo CJ Met proto-oncogene and insulin-like growth factor binding protein 3 overexpression correlates with metastatic ability in well-differentiated pancreatic endocrine neoplasms. Clin Cancer Res (2004) 10:6152–8. 10.1158/1078-0432.CCR-04-0285 15448002

[39] Xi Y Nakajima G Hamil T Fodstad O Riker A Ju J. Association of insulin-like growth factor binding protein-3 expression with melanoma progression. Mol Cancer Ther (2006) 5:3078–84. 10.1158/1535-7163.MCT-06-0424 17172410

[40] Zhong LP Yang X Zhang L Wei KJ Pan HY Zhou XJ Overexpression of insulin-like growth factor binding protein 3 in oral squamous cell carcinoma. Oncol Rep (2008) 20:1441–7. 10.3892/or_00000164 19020726

[41] Sun JM Jun HJ Ko YH Park YH Ahn YC Son YI Insulin-like growth factor binding protein-3, in association with IGF-1 receptor, can predict prognosis in squamous cell carcinoma of the head and neck. Oral Oncol (2011) 47:714–9. 10.1016/j.oraloncology.2011.06.007 21708479

[42] Vestey SB Perks CM Sen C Calder CJ Holly JM Winters ZE. Immunohistochemical expression of insulin-like growth factor binding protein-3 in invasive breast cancers and ductal carcinoma in situ: implications for clinicopathology and patient outcome. Breast Cancer Res (2005) 7:R119–29. 10.1186/bcr963 15642160 PMC1064109

[43] Firth SM Baxter RC. Characterisation of recombinant glycosylation variants of insulin-like growth factor binding protein-3. J Endocrinol (1999) 160:379–87. 10.1677/joe.0.1600379 10076184

[44] Baxter RC. Insulin-like growth factor (IGF)-binding proteins: interactions with IGFs and intrinsic bioactivities. Am J Physiol Endocrinol Metab (2000) 278:E967–76. 10.1152/ajpendo.2000.278.6.E967 10826997

[45] Forbes BE McCarthy P Norton RS. Insulin-like growth factor binding proteins: a structural perspective. Front Endocrinol (2012) 3:38. 10.3389/fendo.2012.00038 22654863 PMC3356058

[46] Payet LD Wang XH Baxter RC Firth SM. Amino- and carboxyl-terminal fragments of insulin-like growth factor (IGF) binding protein-3 cooperate to bind IGFs with high affinity and inhibit IGF receptor interactions. Endocrinology (2003) 144:2797–806. 10.1210/en.2003-0102 12810533

[47] Stover DG Winer EP. Tailoring adjuvant chemotherapy regimens for patients with triple negative breast cancer. Breast (2015) 24(Suppl 2):S132–5. 10.1016/j.breast.2015.07.032 26255198

[48] Tacar O Sriamornsak P Dass CR. Doxorubicin: an update on anticancer molecular action, toxicity and novel drug delivery systems. J Pharm Pharmacol (2013) 65:157–70. 10.1111/j.2042-7158.2012.01567.x 23278683

[49] Buckbinder L Talbott R Velasco-Miguel S Takenaka I Faha B Seizinger BR Induction of the growth inhibitor IGF-binding protein 3 by p53. Nature (1995) 377:646–9. 10.1038/377646a0 7566179

[50] Marzec KA Lin MZ Martin JL Baxter RC. Involvement of p53 in insulin-like growth factor binding protein-3 regulation in the breast cancer cell response to DNA damage. Oncotarget (2015) 6:26583–98. 10.18632/oncotarget.5612 26378048 PMC4694938

[51] Zhang H Cohen AL Krishnakumar S Wapnir IL Veeriah S Deng G Patient-derived xenografts of triple-negative breast cancer reproduce molecular features of patient tumors and respond to mTOR inhibition. Breast Cancer Res (2014) 16:R36. 10.1186/bcr3640 24708766 PMC4053092

[52] Chougule MB Patel AR Jackson T Singh M. Antitumor activity of noscapine in combination with doxorubicin in triple negative breast cancer. PLoS One (2011) 6:e17733. 10.1371/journal.pone.0017733 21423660 PMC3057970

[53] English C Aloi JJ. New FDA-approved disease-modifying therapies for multiple sclerosis. Clin Ther (2015) 37:691–715. 10.1016/j.clinthera.2015.03.001 25846320

[54] Kazandjian D Blumenthal GM Yuan W He K Keegan P Pazdur R. FDA approval of gefitinib for the treatment of patients with metastatic EGFR mutation-positive non-small cell lung cancer. Clin Cancer Res (2016) 22:1307–12. 10.1158/1078-0432.CCR-15-2266 26980062

